# Data Analysis of Physician Competence Research Trend: Social Network Analysis and Topic Modeling Approach

**DOI:** 10.2196/47934

**Published:** 2023-07-19

**Authors:** So Jung Yune, Youngjon Kim, Jea Woog Lee

**Affiliations:** 1 Department of Medical Education Pusan National University Busan Republic of Korea; 2 Department of Medical Education Wonkwang University School of Medicine Iksan Republic of Korea; 3 Intelligence Informatics Processing Lab Chung-Ang University Seoul Republic of Korea

**Keywords:** physician competency, research trend, competency-based education, professionalism, topic modeling, latent Dirichlet allocation, LDA algorithm, data science, social network analysis

## Abstract

**Background:**

Studies on competency in medical education often explore the acquisition, performance, and evaluation of particular skills, knowledge, or behaviors that constitute physician competency. As physician competency reflects social demands according to changes in the medical environment, analyzing the research trends of physician competency by period is necessary to derive major research topics for future studies. Therefore, a more macroscopic method is required to analyze the core competencies of physicians in this era.

**Objective:**

This study aimed to analyze research trends related to physicians’ competency in reflecting social needs according to changes in the medical environment.

**Methods:**

We used topic modeling to identify potential research topics by analyzing data from studies related to physician competency published between 2011 and 2020. We preprocessed 1354 articles and extracted 272 keywords.

**Results:**

The terms that appeared most frequently in the research related to physician competency since 2010 were *knowledge, hospital, family, job, guideline*s, *management,* and *communication*. The terms that appeared in most studies were *education, model, knowledge,* and *hospital*. Topic modeling revealed that the main topics about physician competency included *Evidence-based clinical practice*, *Community-based healthcare*, *Patient care*, *Career and self-management*, *Continuous professional development*, and *Communication and cooperation*. We divided the studies into 4 periods (2011-2013, 2014-2016, 2017-2019, and 2020-2021) and performed a linear regression analysis. The results showed a change in topics by period. The *hot topics* that have shown increased interest among scholars over time include *Community-based healthcare*, *Career and self-management*, and *Continuous professional development*.

**Conclusions:**

On the basis of the analysis of research trends, it is predicted that physician professionalism and community-based medicine will continue to be studied in future studies on physician competency.

## Introduction

### Background

Medical publications began defining competencies in the 1970s [1-3]. Physician competency refers to the essential qualities that a physician should possess. The search for physician competency begins with the question of what it means to be a physician. Competency connotes various ideas, such as which physician traits are desired by society and what supports and promotes this transition of identity. Competency entails the concept of the physician as a professional, what the physician can do, and how the physician approaches their practice [4]. In summary, competency is considered a complex set of behaviors built on the components of knowledge, skills, attitudes, and competence as a personal ability [5]. Competency in this study is the core competency required to successfully perform a physician’s job and includes knowledge, skills, and attitude, regardless of the specific major.

In the medical profession, the competency theme began by pursuing ways for the medical circle to improve the performance of health care workers. The issue arose in response to the growing demands of medical consumers and society as a neoliberalistic ideology spread in the 1970s and consumers’ awareness of their rights increased. In 1972, the American Academy of Pediatrics discussed physician competency by publishing a foundation for evaluating pediatricians’ competency [3]. In 1978, the World Health Organization found the cause of declines in medical service quality to be inadequate education and attempted to improve health care providers’ competencies through Competency-Based Curriculum Development in Medical Education [6]. Since then, this approach has influenced medical education globally, starting with the Royal College of Physicians and Surgeons in Canada and the Accreditation Council for Graduate Medical Education in the United States. Similar programs have been established worldwide, influencing strategies for global human resources and international partnerships for medical training [7]. These include the Outcome Project of the US Accreditation Council for Graduate Medical Education, General Medical Council’s Tomorrow’s Doctors [8-10], Scottish Doctor [11], and Canadian CanMEDS framework [12].

For physicians, competency varies depending on the clinical, cultural, and geographical context [13]. In medical practice, the perception of a medical professional’s competent role changes continuously over time. In the early 19th century, physicians applied ointments and drew blood but did not deliver babies. In the 21st century, physicians are required to use advanced technologies and artificial intelligence in medical surgeries [14,15]. The ability to use technologies required by future health care systems is a challenge for physicians. However, at the same time, communication with patients and colleagues and interprofessional teamwork are essential human skills, and personal traits, such as empathy, humility, compassion, emotional intelligence, and a passion for continuous learning are also emphasized. Varying levels of health care infrastructure over time [16]; social awareness of minority groups [17]; and occasional health care challenges, such as global pandemics [18], emphasize certain specific physician competencies. Physicians’ competency, initially discussed within the scope of their social accountability, includes changes in their roles from the perspective of patients, health care, and self-management [19] of their health and wellness [20]. Thus, physicians’ abilities reflect their social situation and demands. In addition, physicians’ core abilities are expected to change over time. Therefore, this study, which analyzed changes in physician competency by period, will help understand the social demands expected of physicians in each period and identify research topics that should be important in medical education in the future.

To date, studies on physician competency have focused on literature review [21-23] or meta-analyses [24,25]. However, systematic literature reviews have limitations in deriving comprehensively synthesized results because their analyses focus on narrow areas such as subjects, variables, environment, and intervention. In medical education, studies on competency often explore the acquisition, performance, and evaluation of particular skills, knowledge, or behaviors that constitute physician competency. Some of these studies examined patient-physician communication [26], risk management for emergency physicians, technical skills in robotic surgery in urological practice [27], and the instruction of medical staff [28]. Nevertheless, such approaches fail to convey the trends in physician competency research because they explore the essential medical skills for a specific task in a certain context. In addition, people may have different thoughts about the core competencies that physicians should possess. For example, a patient’s expectations of a physician’s ability may differ from a senior physician’s expectation of a junior physician’s ability. Therefore, a more macroscopic method is required to analyze the core competencies of physicians in this era.

Recently, new big data analysis techniques such as social network analysis and topic modeling have been used. These approaches have the advantage of organizing the knowledge structure context by forecasting the trajectory of change in research and future issues and revealing the correlation between concepts. Social network analysis involves detecting influential core keywords in a vast amount of text and showing the relationship between keywords, allowing researchers to comprehend the context intuitively [29,30]. Topic modeling detects hidden topics in text data, analyzes the association and distribution of each topic, and provides integrated information [31]. From a microscopic perspective, it identifies core topics and their relationships. From a macroscopic perspective, it identifies the flow and context of core topics and the trend of topics by period [32]. Text network analysis and topic modeling are ideal approaches for analyzing trends in research on physicians’ competencies and accomplishing research objectives.

### Objectives

The research problems based on the abovementioned research necessity are as follows:

1. Extract core keywords from physician competency studies and create a network
2. Examine the structure and characteristics of the network created based on physician competency studies
3. Examine the main topics through topic modeling in physician competency studies
4. Examine the trend of physician competency studies by topic based on time flow

## Methods

### Data Collection

**Figure 1:**
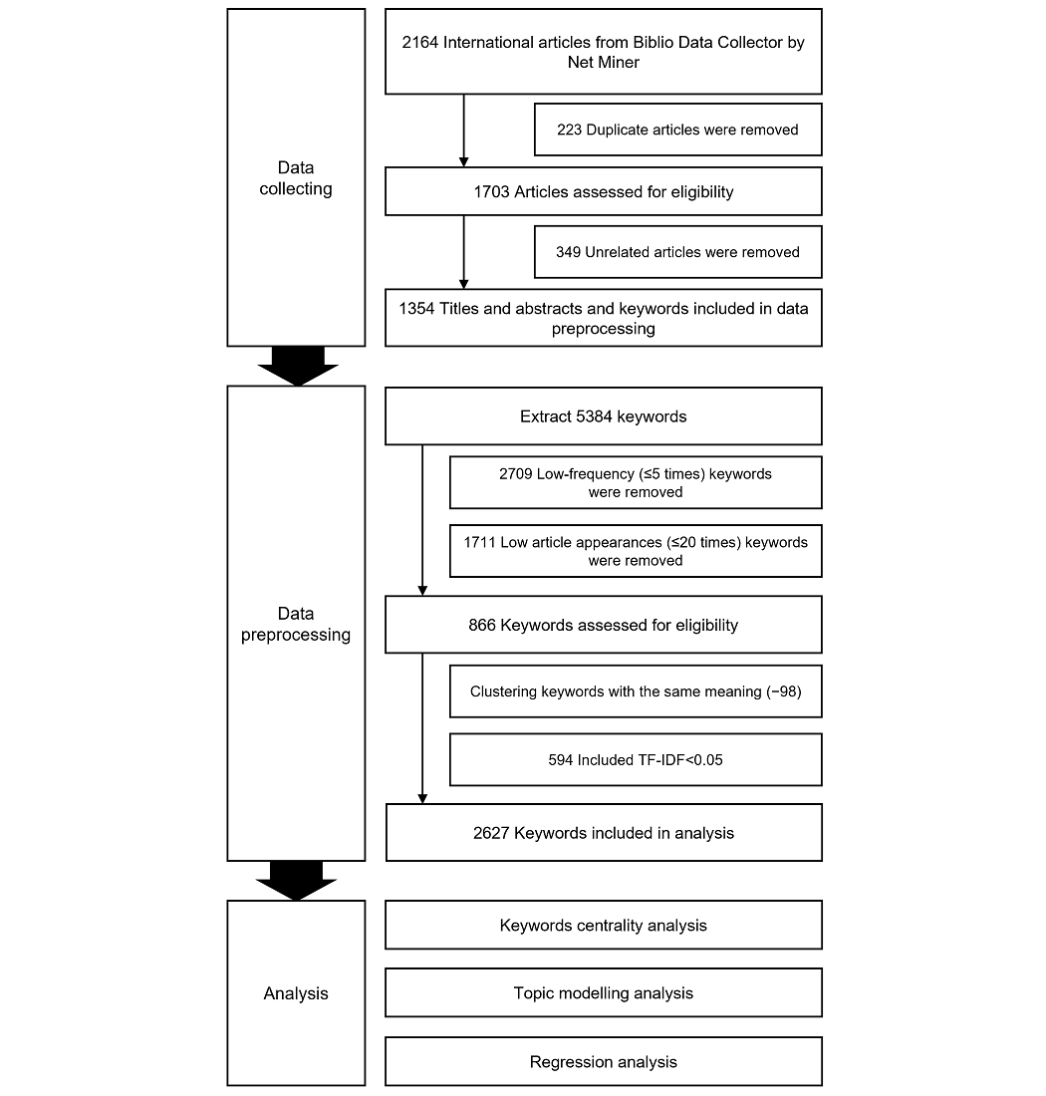
Flow of research procedures. TF-IDF: term frequency–inverse document frequency.

To examine the flow of academic research on physician competency, we selected related research articles as raw data, which were collected using NetMiner Biblio Data Collector (Cyram Inc). We selected 10 years (2011-2020) and collected 2164 articles on physician competency published in *Springer* using the following keywords: “(doctors or physicians) AND ((competence or competency or competencies) or (expertise or expert or proficiency) or (responsibility or accountability or liability or blameworthiness) or (profession or occupation or roles or duties or jobs) or (performance and practice) or professionalism)).” After eliminating 810 articles that overlapped or were unrelated, 1354 articles remained for our analysis. The research flow is illustrated in Figure 1.

### Data Preprocess

Because we could not use the original text in the preselected articles in the analysis, we processed the sentences into separate words as units of analysis. The collected papers consisted of natural language sentences such as theories, knowledge, and opinions. However, the sentences cannot be used directly in the analysis. Therefore, steps must be taken to convert each sentence into an individual word that can be analyzed [33]. In this study, to extract nouns, adjectives, and verbs, we used the morpheme-refining function of the NetMiner program and extracted 5384 words from the titles, abstracts, and keywords of the research articles. Preprocessing was performed to convert these words into analyzable keyword data.

Nouns that were unsuitable for analysis were eliminated. First, we removed words that appeared 5 times or keywords that did not appear in more than 20 of the 1354 articles. In the final stage, we eliminated infrequently appearing words (<5 times) and extremely common words that appeared frequently in all papers (term frequency–inverse document frequency<0.05) [34]. After preprocessing, 272 words were extracted.

### Social Network Analysis

In this study, social network analysis was used to examine the knowledge structure and characteristics through keyword extraction and network generation from physician competency studies, designated as research problems 1 and 2. In addition, the roles of keywords in the network were determined by assessing their importance using social network analysis techniques such as degree centrality, closeness centrality, and betweenness centrality. Social network analysis determines core nodes based on degree centrality, closeness centrality, and betweenness centrality. The degree centrality of a node increases with the number of nodes directly connected to it. Thus, the degree centrality indicates the influence of a node (keyword) based on the number of connected nodes. The betweenness centrality indicates the centrality of a node (keyword) between 2 other nodes (keywords). Betweenness centrality increases when the number of times a node appears on the shortest path between 2 other nodes increases. Keywords with high betweenness centrality control the information flow and exert a substantial influence on the overall connectivity of the network. To visually understand the positions and relationships among keywords, we used spring mapping from NetMiner 4.0. Spring mapping maximizes the characteristics of branching out the graph by placing the connected nodes closer and the disconnected nodes farther, using a simulated annealing technique to balance the 2 forms of distance.

### Topic Modeling

To address research problem 3, we conducted topic modeling to explore the topic areas of physician competency research. Topic modeling was performed using keywords extracted from social network analysis. NetMiner 4.0 was used as the analysis program. Topic modeling is used to predict latent topics based on the association of multiple words in a text. Topic modeling extracts topics from research papers through keyword exploration, which helps recognize knowledge structures and patterns [35]. Knowledge structures are defined as a visualization of keyword clustering and a network map of how concepts in domain knowledge are interrelated [36]. This pattern is defined as the process of change in the knowledge structure over a period [37]. Topic modeling, as a big data analysis technique, provides a quantitative approach for identifying previously undiscovered macrotopic areas in physician competency research. Topics were extracted by applying a latent Dirichlet allocation (LDA) algorithm. The LDA algorithm is a probability model for predicting hidden topics by analyzing the distribution of words observed in a document. It is useful for reducing the data size and producing consistent topics.

**Figure 2:**
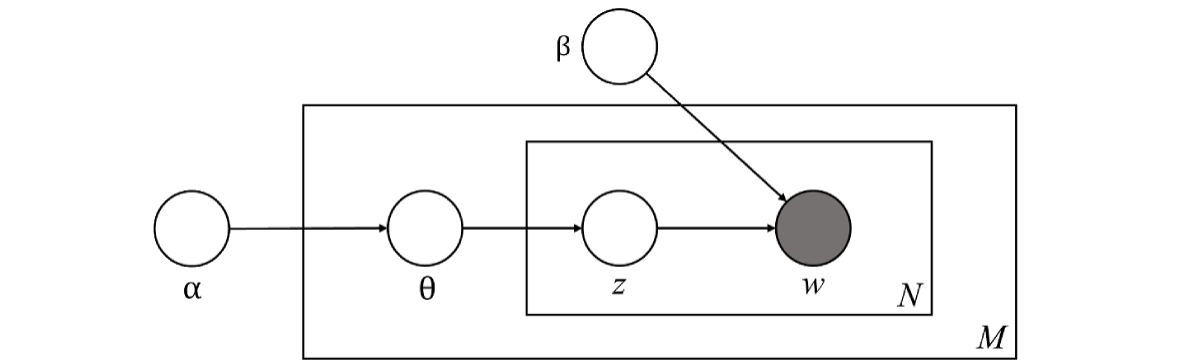
Illustration for the conceptual model of latent Dirichlet allocation. α: a parameter that represents the Dirichlet prior for the document topic distribution; β: a parameter that represents the Dirichlet for the word distribution; θ: a vector for topic distribution over a document d; z: a topic for a chosen word in a document; w: specific words in N; M: document length; N: number of words in the document.

Figure 2 shows a graphical model of LDA. The boxes in the figure are “plates,” which represent duplicates. The outer plate represents the document (M) and the inner plate represents repeatedly selected topics (z) and words (w) within the document (N). “ϴ” indicates the distribution of topics in the document. Both “α” and “β” are hyperparameters that indicate Dirichlet distribution. LDA cannot be directly used to determine the number of topics, so the third hyperparameter is the “number of topics” the algorithm will discover. The probability calculation formula is as follows [38]:



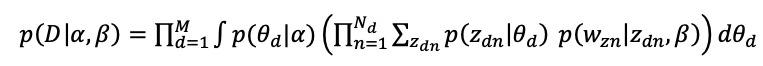



To increase the accuracy of the results, appropriate Cronbach α values, β values, number of topics, and keywords should be determined [39]. To evaluate how well each keyword described each topic, we used one of the topic consistency metrics, the silhouette coefficient. The silhouette coefficient is an indicator that evaluates how well keywords, which are components of a topic, are classified [40]. A value closer to 1 indicates that the keywords within each cluster are well formed. Moreover, good clustering means that similarity to other topics is low and keywords within the same cluster describe a topic well. For topic *t* characterized by a higher-order word set *W_t_* (any word whose probability exceeds a predefined threshold or a fixed number of high-order words), the consistency formula is defined as follows [41]:



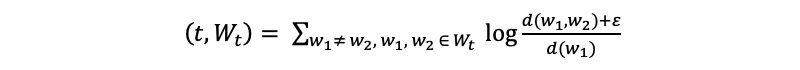



In this study, we set the term frequency–inverse document frequency threshold value at 0.5 and word length at 2. We used a silhouette coefficient to calculate the optimal values for Cronbach α, β, and the number of topics. A silhouette coefficient (or score) closer to 1 had higher explanatory power, validating Cronbach α*,* validating β, and the number of topics and descriptions of the object in the topic model were well matched. We also used a silhouette-clustering configuration. To determine the optimal number of topics, we conducted a comprehensive analysis by varying the number of topics from 5 to 30 and exploring Cronbach α values ranging from .01 to .99 as well as β values ranging from .01 to .99. The silhouette coefficient was used as the evaluation criterion. Our findings revealed that the highest silhouette coefficient of 0.782 was achieved using a Cronbach α value of .89, a β value of .97, and 6 topics. Subsequently, we proceeded with topic modeling using the identified parameters.

### Analysis of Change in Topics by Period

To address research problem 4—the change in topics in physician competency research over time—the analysis was divided into 4 periods: 2011-2013, 2014-2016, 2017-2019, and 2020-2021. We divided the period into before and after the COVID-19 outbreak, and the researchers discussed and classified them. Subsequently, we analyzed how the percentages of each topic changed. To categorize the topics by checking the pattern of increased or decreased topics by period, we performed a linear regression analysis using SPSS (version 23.0; IBM Corp). We used the categorized periods as independent variables, and the percentage of each topic as the dependent variable. Following the linear regression analysis, we classified the keywords into 4 types based on the regression coefficient sign (+ or −) and the significance probability (*P* value): hot, warm, cool, and cold. If the coefficient is positive and the significance probability is ≤.05, it is classified as a “hot topic” with increasing research interest. Conversely, if the coefficient is negative and the significance probability is ≤.05, it is classified as a “cold topic” with decreasing research interest. Meanwhile, the clusters that were either positive or negative with no statistical significance and with a significance probability of ≥.05 were classified as “warm” and “cool” topics, respectively [42].

### Ethics Approval

This study was conducted after obtaining approval from the Medical Research Ethics Review Committee of Chungnam National University Hospital (CNUH 2021-02-025).

## Results

### Core Keywords From Physician Competency Studies

Between 2011 and 2020, the words that appeared most frequently in physician competency studies were *knowledge* (604 times), *hospital* (598 times), *family* (597 times), *job* (573 times), *guideline* (491 times), *management* (482 times), and *communication* (443 times). The words that appeared in most studies were *education* (n=256), *model* (n=243), *knowledge* (n=238), and *hospital* (n=234). Table 1 presents the 25 words with the highest frequency and the number of articles in which they appeared.

**Table 1:** High-ranking keywords by frequency in research.

Rank	Keyword	Frequency, n	Articles in which it appears, n
1	Knowledge	604	238
2	Hospital	598	234
3	Family	597	161
4	Job	573	111
5	Guideline	491	164
6	Management	482	206
7	Communication	443	154
8	Education	437	256
9	Model	425	243
10	Assessment	407	150
11	Attitude	388	151
12	Information	381	213
13	Health care	379	196
14	Experience	368	224
15	Medication	356	64
16	Intervention	332	152
17	Cancer	330	89
18	Disease	313	150
19	Change	276	147
20	Need	268	196
21	Development	255	170
22	Behavior	253	87
23	Barrier	238	97
24	Evidence	234	158
25	Practice	227	199

### Social Network Analysis

**Figure 3:**
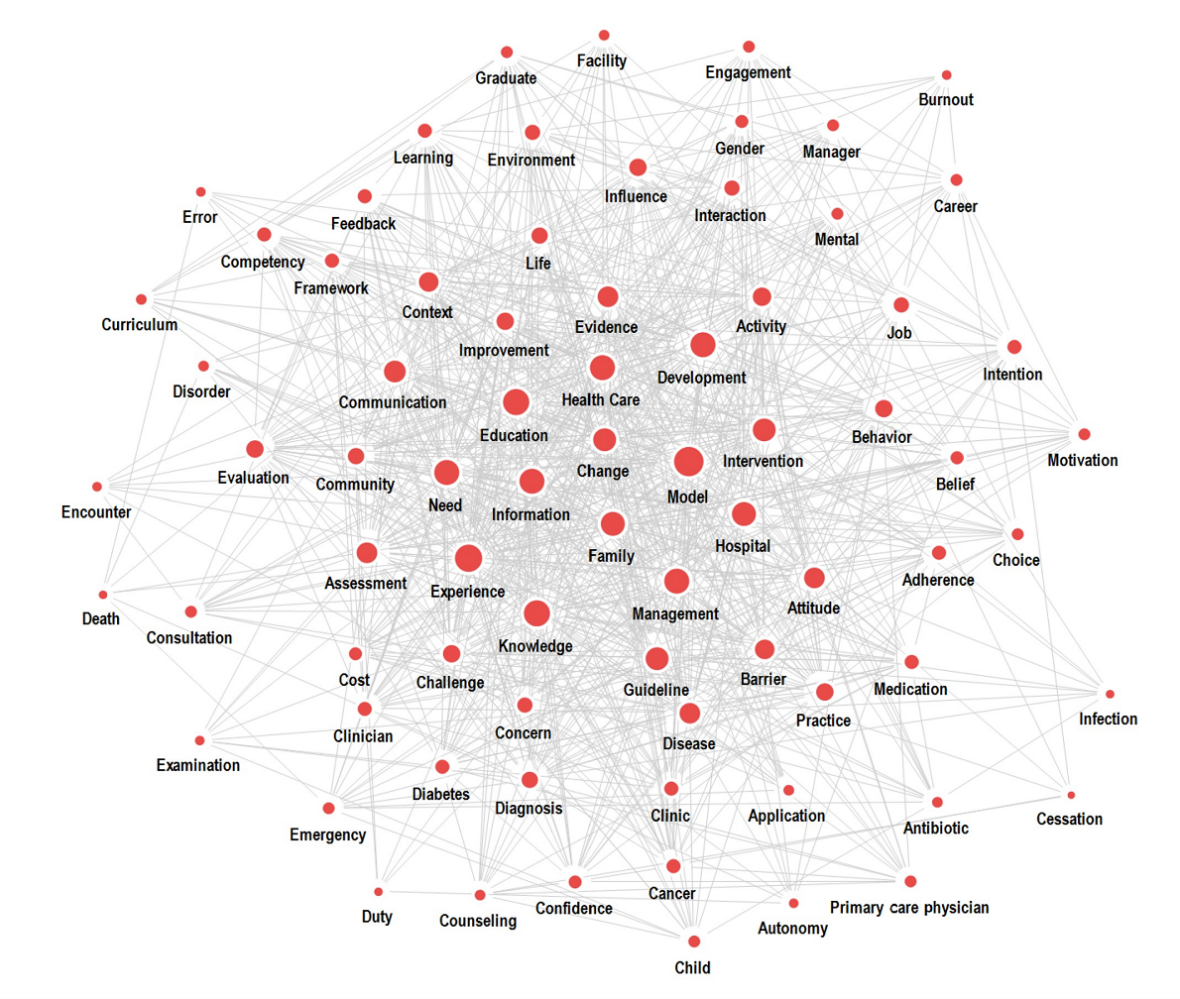
Centrality network by the 2D spring network map.

Table 2 presents keyword degree, closeness, and betweenness centrality from physician competency studies. Each keyword and its degree are as follows. The higher the degree of the keyword, the stronger the influence in the network. The keywords with the highest degree and closeness centrality were, in order, *model* (0.988 and 0.988, respectively), *education* (0.981 and 0.982), *experience* (0.973 and 0.975), *health care* (0.973 and 0.975), *hospital* (0.973 and 0.975), and *information* (0.973 and 0.975). The keywords with the highest betweenness centrality were, in order, *model* (0.007), *education* (0.006), *knowledge* (0.006), *management* (0.006), and *education* (0.006). In total, 29 words belonged to the top 30 keywords in all 3 centrality types. The keywords with a high degree centrality were also high in closeness centrality. However, the betweenness centrality of environment was higher than the degree and closeness centrality. Figure 3 shows the network map for centrality.

**Table 2:** High-ranking keywords by degree, closeness, and betweenness centrality.

Rank	Degree centrality		Closeness centrality			Betweenness centrality	
	Keyword	Degree	Keyword	Degree	Keyword		Degree
1	Model	0.98881	Model	0.98893	Model		0.00651
2	Education	0.98134	Education	0.98169	Education		0.00630
3	Experience	0.97388	Experience	0.97455	Knowledge		0.00616
4	Health care	0.97388	Health care	0.97455	Information		0.00616
5	Hospital	0.97388	Hospital	0.97455	Management		0.00614
6	Information	0.97388	Information	0.97455	Experience		0.00614
7	Knowledge	0.97388	Knowledge	0.97455	Hospital		0.00614
8	Management	0.97388	Management	0.97455	Need		0.00610
9	Need	0.97388	Need	0.97455	Health care		0.00607
10	Development	0.96269	Development	0.96403	Development		0.00600
11	Evidence	0.95522	Evidence	0.95714	Evidence		0.00571
12	Change	0.95149	Change	0.95374	Change		0.00570
13	Intervention	0.94776	Intervention	0.95036	Intervention		0.00551
14	Family	0.93657	Family	0.94035	Disease		0.00543
15	Disease	0.93284	Disease	0.93706	Family		0.00542
16	Guideline	0.92910	Guideline	0.93380	Guideline		0.00512
17	Practice	0.92164	Practice	0.92734	Practice		0.00509
18	Assessment	0.91418	Assessment	0.92096	Assessment		0.00502
19	Challenge	0.91418	Challenge	0.92096	Communication		0.00493
20	Attitude	0.91045	Attitude	0.91781	Challenge		0.00492
21	Communication	0.90672	Communication	0.91468	Attitude		0.00480
22	Evaluation	0.90299	Evaluation	0.91157	Evaluation		0.00474
23	Concern	0.88806	Concern	0.89933	Concern		0.00456
24	Influence	0.88060	Influence	0.89333	Influence		0.00451
25	Activity	0.86940	Activity	0.88449	Improvement		0.00442
26	Improvement	0.86940	Improvement	0.88449	Barrier		0.00430
27	Community	0.86194	Community	0.87869	Activity		0.00427
28	Barrier	0.85821	Barrier	0.87582	Environment		0.00409
29	Implementation	0.85821	Implementation	0.87582	Implementation		0.00408
30	Cost	0.85448	Cost	0.87296	Community		0.00406

### Topic Modeling

Regarding the number of topics from physician competency studies, we decided on 6 topics (silhouette=0.782) by considering the silhouette coefficient and the validity of interpretation. The core keywords by topic are listed in Table 3. The top keywords in topic 1 were *management*, *intervention*, *disease*, *cost*, and *medication*. The top keywords in topic 2 were *family*, *health care*, *information*, *community*, and *need*. The high-ranking keywords in topic 3 were *knowledge*, *attitude*, *cancer*, *guideline*s, and *barrier*s. The high-ranking keywords in topic 4 were, in order, *hospital*, *job*, *burnout*, *model*, *gender*, and *intention*. The top keywords in topic 5 were, in order, *assessment*, *education*, *competency*, *development*, and *graduation*. Finally, the high-ranking keywords in topic 6 were, in order, *communication*, *consultation*, *experience*, *emergency*, and *feedback*.

**Figure 4:**
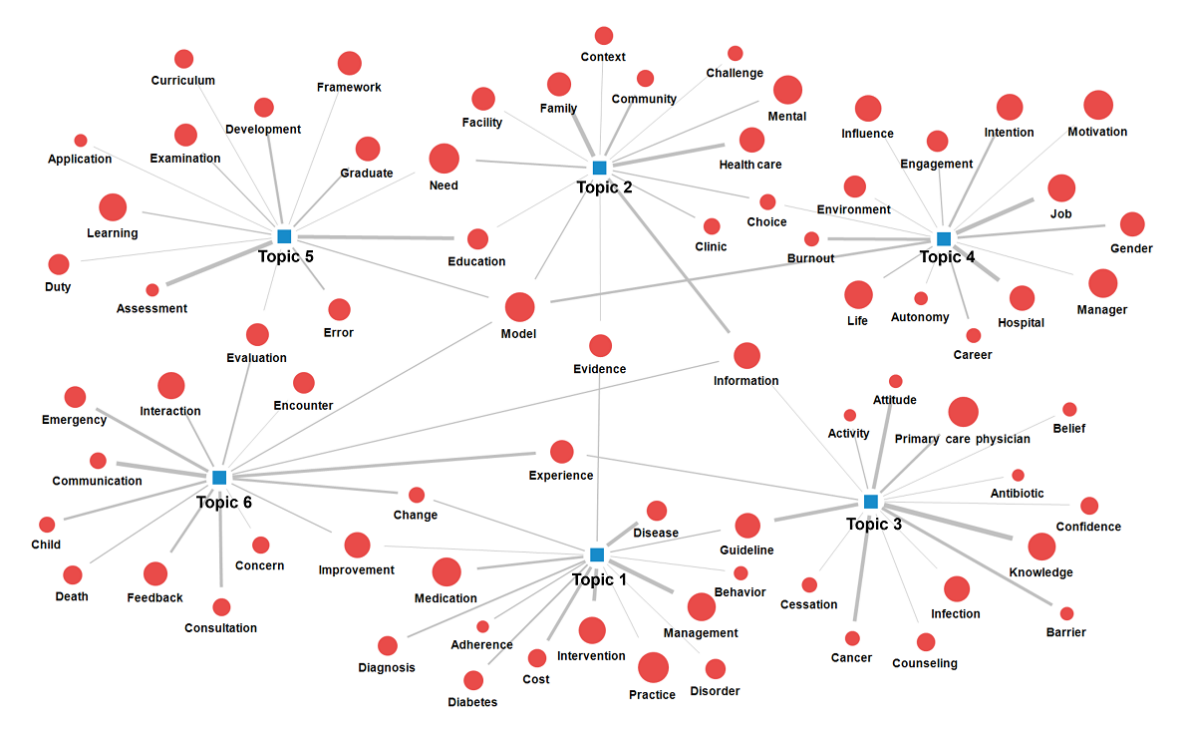
Topic modeling network: topic-keyword map.

Topic groups were labeled based on high-ranking core keywords in terms of probability distribution: topic 1, *Evidence-based clinical practice*; topic 2, *Community-based healthcare*; topic 3, *Patient care*; topic 4, *Career and self-management*; topic 5, *Continuous professional development*; and topic 6, *Communication and cooperation*. On the basis of the nature of the topics, we divided the topic groups into 2 domains: those related to job competency and those related to personal competency. The job domain includes topics 1, 2, 3, and 6, and the personal domain includes topics 4 and 5. Figure 4 presents the results of visualizing the 7 networks of core keywords using a topic-keyword map.

**Table 3:** Core keywords by topic.

Rank	Domain and topic						
	Job					Personal	
	Evidence-based clinical practice	Community-based healthcare	Patient care	Communication and cooperation	Career and self-management		Continuous professional development
1	Management	Family	Knowledge	Communication	Hospital		Assessment
2	Intervention	Health care	Attitude	Consultation	Job		Education
3	Disease	Information	Cancer	Experience	Burnout		Competency
4	Cost	Community	Guideline	Emergency	Model		Development
5	Medication	Need	Barrier	Feedback	Gender		Graduate
6	Diagnosis	Choice	PCP^a^	Child	Intention		Error
7	Diabetes	Clinic	Activity	Change	Career		Learning
8	Adherence	Model	Experience	Interaction	Life		Examination
9	Guideline	Mental	Confidence	Evaluation	Engagement		Model
10	Change	Education	Counseling	Model	Manager		Framework

^a^PCP: primary care provider.

### Core Topics by Period

Table 4 presents the changes in topics over time and Table 5 lists the rate of each topic during this period. Over the past 10 years, each topic has been studied with similar weights. Communication and cooperation accounted for 18.61% (252/1354) of the articles, followed by Evidence-based clinical practice at 17.73% (240/1354) and Continuous professional development at 16.77% (227/1354). Regarding the domains, studies related to physicians’ job competencies accounted for 61.21% (910/1354), whereas those related to physicians’ personal competencies accounted for 32.79% (444/1354).

**Table 4:** Number of research on 6 topics by year (n=1354).

Domain and topic		2011 (n=82)	2012 (n=107)	2013 (n=98)	2014 (n=152)	2015 (n=134)	2016 (n=120)	2017 (n=125)	2018 (n=141)	2019 (n=129)	2020 (n=167)	2021 (n=99)	Total (n=1354)
**Job, n (%)**													
	Evidence-based clinical practice	15 (6.3)	24 (10)	24 (10)	27 (11.3)	22 (9.2)	21 (8.8)	25 (10.4)	26 (10.8)	25 (10.4)	21 (8.8)	10 (4.2)	240 (100)
	Community-based healthcare	11 (5.2)	13 (6.1)	13 (6.1)	22 (10.3)	26 (12.2)	22 (10.3)	17 (8)	18 (8.5)	20 (9.4)	29 (13.6)	22 (10.3)	213 (100)
	Patient care	13 (6.3)	22 (10.7)	6 (2.9)	29 (14.2)	12 (5.9)	15 (7.3)	18 (8.8)	23 (11.2)	14 (6.8)	36 (17.6)	17 (17.6)	205 (100)
	Communication and cooperation	20 (7.9)	21 (8.3)	22 (8.7)	25 (9.9)	30 (12)	21 (8.3)	22 (8.7)	21 (8.3)	25 (9.9)	24 (9.5)	21 (8.3)	252 (100)
**Personal, n (%)**													
	Career and self-management	11 (5.1)	13 (6)	16 (7.4)	25 (11.5)	19 (8.8)	22 (10.1)	20 (9.2)	24 (11.1)	26 (12)	30 (12)	11 (13.8)	217 (100)
	Continuous professional development	12 (5.3)	14 (6.2)	17 (7.5)	24 (10.5)	25 (11)	19 (8.4)	23 (10.1)	29 (12.8)	19 (8.4)	27 (12)	18 (7.9)	227 (100)

**Table 5:** The topic frequency and possession during the period (n=1354).

Domain and topic		Period 1: 2011-2013 (n=287)	Period 2: 2014-2016 (n=406)	Period 3: 2017-2019 (n=395)	Period 4: 2020-2021 (n=266)	Total (n=1354)
**Job, n (%)**						
	Evidence-based clinical practice	63 (22)	70 (17.2)	76 (19.2)	31 (11.6)	240 (17.7)
	Community-based healthcare	37 (12.9)	70 (17.2)	55 (13.9)	51 (19.2)	213 (15.7)
	Patient care	41 (14.2)	56 (13.8)	55 (13.9)	53 (19.9)	205 (15.1)
	Communication and cooperation	63 (225)	76 (18.7)	68 (17.2)	45 (16.9)	252 (18.6)
**Personal, n (%)**						
	Career and self-management	40 (13.9)	66 (16.3)	70 (17.7)	41 (15.4)	217 (16)
	Continuous professional development	43 (15)	68 (16.8)	71 (18)	45 (16.9)	227 (16.8)

Dividing the period into 3-year groups, *Evidence-based clinical practice* (63/240, 26.3%) and *Communication and cooperation* (37/213, 12.9%) were studied most often during the first period (2011-2013), whereas research on *Community-based healthcare* (41/205, 14.3%) and *Career and self-management* (40/217, 13.9%) was conducted on a small scale. *Communication and cooperation* was studied most often during the second period (2014-2016), but the weight decreased compared with the first period (from 22% to 18.7%).

The topics that increased in weight compared with the first period were *Career and self-management* (from 13.9% to 16.3%) and *Continuous professional development* (from 15% to16.8%). During the third period (2017-2019), *Evidence-based clinical practice* (76/240, 19.2%) was studied the most, followed by *Continuous professional development* (71/227, 18%) and *Career and self-management* (70/217, 17.7%). During the fourth period, which covers the COVID-19 pandemic (2020-2021), *Patient care* (53/205, 19.9%) and *Community-based healthcare* (51/213, 19.2%) were the most studied. Compared with the third period, *Patient care* increased from 13.9% to 19.9%, and *Community-based healthcare* increased from 13.9% to 19.2%. Conversely, there were decreases in *Evidence-based clinical practice* (from 19.2% to 11.6%), *Career and self-management* (from 17.7% to 15.4%), and *Continuous professional development* (from 18% to 16.9%).

Regarding the overall possession during the first 3 periods before the COVID-19 pandemic, there was increased research interest in *Career and self-management* (from 13.9% to 16.3% to 17.7%) and *Continuous professional development* (from 15% to 16.8% to 18%) but decreased interest in *Communication and cooperation* (from 22% to 18.7% to 17.2%). In the fourth period, during the COVID-19 pandemic, there was increased research interest in *Community-based healthcare* (45/252, 16.9%) and *Patient care* (53/502, 19.9%) but decreased interest in *Evidence-based clinical practice* (31/240, 11.6%), *Career and self-management* (41/217, 15.4%), and *Continuous professional development* (45/227, 16.9%).

In terms of domains, studies on physicians’ personal competencies increased from the first to the third period (from 28.9% to 33% to 35.7%, respectively). However, it decreased to 32.3% between 2020 and 2021 after the COVID-19 outbreak. Studies on physicians’ job competency gradually decreased (from 71.2% to 67.8% to 64.3%), before increasing to 67.8% during the fourth period.

### Topic Characteristics by Period

**Figure 5:**
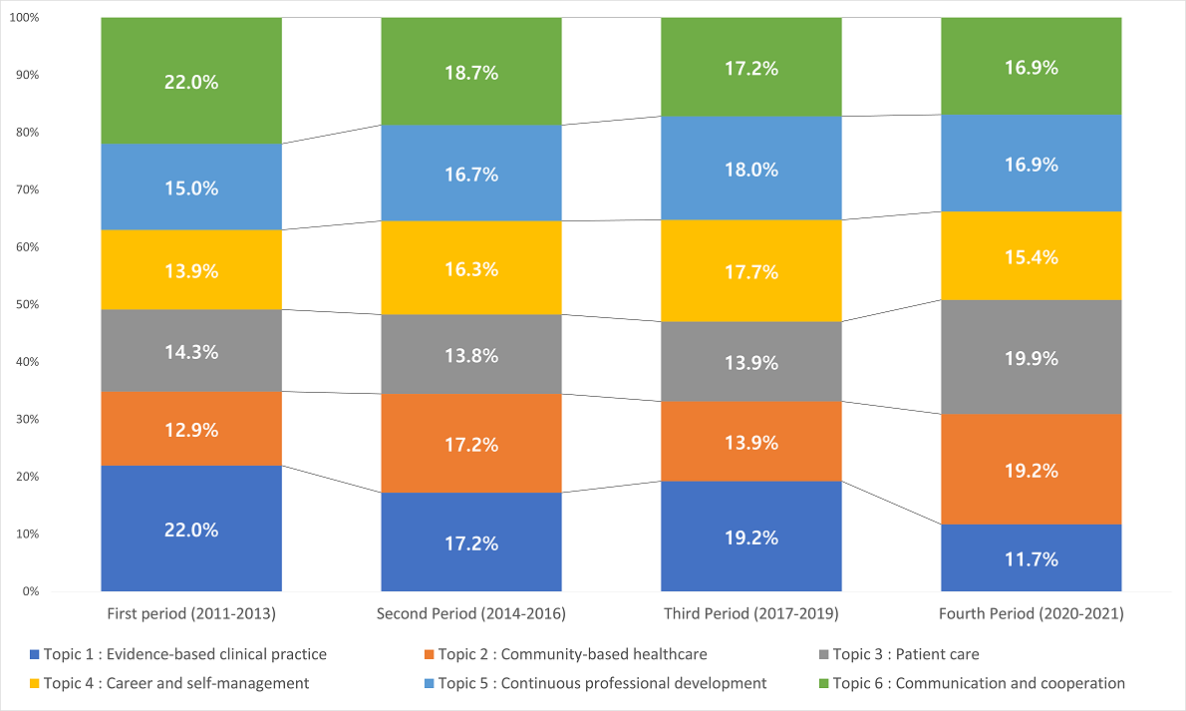
Topic trend during the period.

Table 6 presents the topic characteristics for each period. We performed linear regression analysis to examine the characteristics of the 6 topics. Three topics were classified as *hot topics* with a positive regression coefficient and statistical significance: topic 2 (B=1.315; *t_9_*=2.621; *P*=.03), topic 4 (B=1.758; *t_9_*=5.414; *P*=.001), and topic 5 (B=1.339; *t_9_*=2.963; *P*=.02). We found no *cool* (negative regression coefficients and no statistical significance) or *cold* topics (negative regression coefficients and no statistical significance). Figure 5 shows the subject possession during this period.

**Table 6:** Regression analysis results for each topic.

Domain and topic		B	β	*t* test (*df*)	*P* value	Durbin-Watson statistic	Topic type
**Job**							
	Evidence-based clinical practice	0.388	.339	1.019 (9)	.34	1.535	—^a^
	Community-based healthcare	1.315	.680	2.621 (9)	.03	1.181	Hot
	Patient care	1.248	.426	1.331 (9)	.22	2.960	—
	Communication and cooperation	0.248	.251	0.733 (9)	.48	1.775	—
**Personal**							
	Career and self-management	1.758	.886	5.414 (9)	.001	2.013	Hot
	Continuous professional development	1.339	.723	2.963 (9)	.02	2.157	Hot

^a^Not available.

## Discussion

### Principal Findings

In this study, we used social network analysis to examine keywords and their relationships in physician competency studies conducted over the past 10 years. Topic modeling identified the top 5 research topics, visualized the relationships among them, and described the research possession over time.

Discussions on physicians’ competency arose in the 1990s because of the social atmosphere of consumerism, which demands accountability in all aspects of the profession. The medical and health care field emphasizes physicians’ roles and attitudes, reflecting the demands of medical consumers, such as citizens [43] and local communities [44]. Educational institutions have received demands to improve the curriculum considering educational outcomes [4,45]. Since the 1990s, some countries have begun to define the competence of their physicians and specify their components [46]. After the “Project on the future global role of the physician in healthcare” of the World Federation for Medical Education in 2012, it has become more active in many countries [7]. As a goal of the medical community, a competency model for desirable physicians was constructed in the 2000s, centered on Canada [46], the United States [47], the United Kingdom [48], and Scotland [11]. Since then, scholars have actively studied the development of the curriculum and revision of content, reflecting this competency model [8,49,50]. The physician competency studies conducted since 2010, which we analyzed, are on the continuum of physician competency studies carried out over the past 20 years in a large framework.

The words that appeared most frequently in physician competency studies since 2010 are, in order, *knowledge*, *hospital*, *family*, *job*, *guideline*, *management*, and *communication*. The words that appeared in most studies were *education*, *model*, *knowledge*, and *hospital*.

Previously, a physician’s professional competence was defined as the habitual and careful use of communication, knowledge, skills, clinical reasoning, emotions, values, and reflection in everyday care for the benefit of individuals and communities [51]. The keywords frequently used in this study had a broad coverage, including the knowledge and context necessary for physicians to perform medical practice, a standardized framework necessary to meet social needs, and traits pertaining to physician groups or individuals.

In the network of top keywords, the keywords with high degree and closeness centrality values were *model*, *education*, *experience*, *health care*, and *hospital*s. Keywords with high betweenness centrality values were *model*, *education*, and *knowledge*. The keyword that emerged in the centrality analysis was *change*. Degree centrality indicates the number of times a node appears simultaneously with other nodes in a network. Closeness centrality connotes the distance between nodes within a network, and betweenness centrality connotes the role of a *bridge* between different nodes within a network. Network centrality analysis of keywords revealed that the 2 central nodes leading physician competency studies over the past 10 years were *model* and *education*. *A model* refers to a pattern, plan, or demonstration that illustrates the structure or work of an object, system, or concept. In competency studies, *model* began with a core competency model that explained what constitutes a physician. Later, scholars focused on different models essential for medical practice, such as specific clinical contexts, resident training, patient-physician communication, leadership [52], and health care management.

Competency-based education for medical students and residents refers to an educational method that uses the content or criteria derived from the previous competency model. In other words, a professional’s competency should be built gradually based on scientific knowledge, basic clinical skills, and moral development. Education and experience are essential for acquiring or maintaining expertise in a professional. Song et al [53] conducted a bibliometric analysis of medical expertise from 2010 to 2019. According to these studies, academic journals on medical education primarily include studies on expertise. Likewise, studies on physician competency over the past 10 years have examined methods for developing, implementing, and evaluating competency through education.

Through topic modeling, we identified 6 latent topics. Topic modeling is a researcher-centered content analysis method that identifies a specific pattern assumed to be latent in a document or text and derives a potentially meaningful topic. We also set topic names based on keywords derived from this study. On the basis of relevance of the topic, we divided it into 2 domains: job and personal competency. The job domain comprises *Evidence-based clinical practice*, *Community-based healthcare*, *Patient care*, and *Communication* *and cooperation*, whereas the personal domain comprises *Career and self-management* and *Continuous professional development*. The topics derived from topic modeling in this study cover the criteria for physician competency suggested in many countries. In Canada, the Canadian Medical Education Directions for Specialists [46] organized physician roles into 7 competencies: medical experts, communicators, collaborators, leaders, health advocators, scholars, and professionals. In the United Kingdom, “Good Medical Practice 2020” [54] describes physician competency in terms of 4 core competencies: knowledge, skill, and performance; safety and quality; communication, partnership, and teamwork; and trust maintenance. In the United States, the Accreditation Council for Graduate Medical Education laid out 6 core competencies [46] focusing on the areas of physician activities: patient care, professionalism, interpersonal and communication skills, medical knowledge, systems-based practice, and practice-based learning and improvement.

They also comprise the complex knowledge, skills, and attitudes that physicians must possess. Previous studies have emphasized the topics of physician competency research resulting from this study [5]. First, *Evidence-based clinical practice* is a core competency required for clinicians. It provides a framework for integrating research evidence into health care delivery [55], including patient history taking and analysis, physical examination, and diagnostic accuracy. The implementation of evidence-based practice principles has resulted in notable advances in improving the quality of delivered health care [56]. In addition, over the last 20 years, evidence-based practice has been increasingly integrated as a core component of undergraduate, postgraduate, and continuing education health programs worldwide [12]. Second, *Community-based healthcare* is increasingly emphasized in terms of the need for improvement from a broad cultural and institutional perspective to improve the quality of medical care [57]. *Community-based healthcare* has recently attracted more attention, as primary care is emphasized to solve social problems such as population aging and COVID-19 [58].

Third, the topic of *Patient care* means patient-centered care (PCC). PCC enhances health outcomes, such as improved patient satisfaction, behavior change, trust, patient adherence, providers’ clinical accuracy, disease management plans, and active patient self-management [59]. Therefore, PCC is a crucial attribute of high-quality health care services [60]. Fourth, physicians’ interpersonal and communication skills have a signiﬁcant impact on patient care. Furthermore, it is correlated with improved health outcomes and quality [61]. Ineffective communication skills are associated with malpractice claims and suits [62] and medication errors [63]. Communication is a core clinical skill that can be taught and learned [64]. Moreover, interprofessional communication skills are essential competencies for medical students to become physicians [65].

Fifth, among the 2 topics belonging to the personal domain, *Career and self-management* is related to physicians’ burnout. Job satisfaction can affect physicians’ physical and mental illnesses, such as depression and burnout [66], and is related to patient safety and quality of care [67]. In addition, burnout syndrome is a major concern in occupational health [55]. Therefore, organizations should emphasize the importance of physicians’ self-care (rest, a healthy lifestyle, breaks, and sufficient sleep) and regular burnout screening [68]. Finally, *Continuous professional development* refers to the attitude of lifelong learning as a professional. Through professional development such as lifelong learning, medical specialists maintain their professional competence in addition to keeping track of and gaining advancing knowledge [69]. Furthermore, continuing professional development and lifelong learning are crucial for securing high-quality health care, patient safety, and societal trust in the health care system [70]. In other words, the subdomains of physician competency emphasized in many countries have been the main research topics over the past 10 years. Specifically, the main research topics include *Evidence-based medicine* (an explicit means for generating an important answerable question, interpreting new knowledge, and judging how to apply that knowledge in a clinical setting) [71]; *Community-based healthcare* (emphasizes social responsibility); *Patient care* (considering the patient’s condition and circumstances throughout the treatment process); *Communication and cooperation* with patients, families, and colleagues; *Career and self-management*; and *Continuous professional development* for maintaining competency as scholars and professionals.

An examination of the changes in research topics over the past 10 years revealed that more studies have been conducted on job competencies than on physicians’ personal competencies. Personal domain studies gradually increased from the first to the third period; however, after the COVID-19 outbreak (2020-2021), the number of job domain studies increased.

The most studied topic was *Communication and cooperation*. The topics that showed an increasing frequency and possession before the COVID-19 outbreak (first to third period) were *Career and self-management* and *Continuous professional development*; studies on *Communication and cooperation* showed a decreasing frequency and possession. Studies on *Evidence-based clinical practice* have also gradually decreased (first through second through fourth periods), except during the third period.

Most studies conducted before the COVID-19 outbreak covered physicians’ individual professionalism [10,72]. However, during the COVID-19 pandemic (fourth period), studies on *Community-based healthcare* and *Patient care* increased. This can be explained as follows: physicians’ social responsibility and community-centered care began to be emphasized during the COVID-19 pandemic, and the importance of care centered on patients and communities has resurfaced. Particularly during the COVID-19 pandemic, many problems threatened patients’ health because of the gap in health and medical care, despite individual physicians’ expertise and commitment. These challenging social situations highlight the importance of *Community-based healthcare* [73,74].

In times of crisis, the role of physicians can be broadened. For example, physicians have a duty not only to take care of their patients but also to protect them from infection, and thus take care of their families [18]. In addition, social interventions, such as school closures, affect the supply and demand for medical personnel. However, it is not easy to clarify whether a physician’s role in situations such as COVID-19 is regular duty. Nevertheless, COVID-19 has broadened the demand for physicians’ roles and competencies. This was also manifested in “hot topics” that have gradually increased over the past 10 years, such as *Community-based healthcare*, *Career and self-management*, and *Continuous professional development*. This indicates that topics related to *Community-based healthcare* are gradually becoming more important [73,74], as are topics related to the professionalism of individual physicians [10,51,53,72].

On the basis of the results of this study, the keywords that many researchers were interested in over the past 10 years were *model* and *education*. Therefore, they developed competency-based education and training systems at the hospital, university, and national levels. Consequently, many countries and training institutions, such as hospitals and universities, have developed and educated physicians with competency-based curricula. This was effective in cultivating physicians’ competency in responding to social needs.

The topics we should pay attention to are *Community-based healthcare*, *Career and self-management*, and *Continuous professional development*, which are research topics that have gradually increased with time. This indicates that the scope of physicians’ competencies has been studied more extensively and comprehensively than in the past. It is meaningful in that it defines physicians’ competency as the ability to develop into professional and social leaders, beyond just the ability necessary to perform a job.

The general public’s and patients’ expectations and consciousness of medical care are changing, and the medical system pursued by society is also changing. Consequently, perceptions of the roles of medicine and physicians are rapidly evolving. In line with these changes, research on the core competencies of physicians must be conducted using more detailed competencies and major fields.

### Limitations

This study had some limitations. First, it was difficult to repeat the keyword refining process in the keyword network analysis. To eliminate researcher subjectivity, we have described the analytical procedure in this study. Second, the study period was not equally divided. The last period is short, covering only 6 months, between 2020 and 2021. Because we forecasted that the effect of the COVID-19 pandemic since December 2019 would be reflected between 2020 and the first half of 2021, we set these periods separately. Considering the gap between the time of undertaking and publishing this study, it is unreasonable to argue that the last period accurately reflected the pandemic. However, we believe that it is worth examining the effect of the pandemic because of its global nature, which surpasses its regional and cultural characteristics.

Third, similar to other studies, we explored articles published in English. Physician competency studies are influenced by cultural and social demands and changes in the context of medical services. Although we did not identify the regions in which the studies were published, similar studies on medical professionalism [52] and medical education [75] led us to believe that the English publications in this study were from North America, Europe, or parts of Asia. Future studies should analyze the differences in research trends in physician competency based on culture and region. Finally, this study investigated the core competencies for successfully performing a physician’s job, regardless of the specific major. Subsequently, we can research physician’s competencies for each major.

### Conclusions

The top research topics on physician competency over the past 10 years are *Evidence-based clinical practice*, *Community-based healthcare*, *Patient care*, *Career and self-management*, *Continuous professional development*, and *Communication and cooperation*. The discussion of physician competency entails the establishment of a physician’s fundamental roles and competencies based on a constantly changing health care environment and the implementation of education from studying to competency acquisition. Studies on competency include discussions on the model physician desired by society, as well as the issue of wellness encompassing an individual physician’s job choice and quality of life.

The hot topics in physician competency studies conducted within the past 10 years are *Community-based healthcare* in the job domain and *Career and self-management* and *Continuous professional development* in the physician’s personal domain. These 2 areas are hot topics that have gradually gained interest over time.

## Abbreviations

LDA: latent Dirichlet allocation
PCC: patient-centered care
