# Correction: Data Analysis of Physician Competence Research Trend: Social Network Analysis and Topic Modeling Approach

**DOI:** 10.2196/53484

**Published:** 2023-10-31

**Authors:** So Jung Yune, Youngjon Kim, Jea Woog Lee

**Affiliations:** 1 Department of Medical Education, Pusan National University Busan Republic of Korea; 2 Department of Medical Education, Wonkwang University School of Medicine Iksan Republic of Korea; 3 Intelligence Informatics Processing Lab Chung-Ang University Seoul Republic of Korea

In “Data Analysis of Physician Competence Research Trend: Social Network Analysis and Topic Modeling Approach” (JMIR Serious Games 2023;11(1):e47934) the authors made one correction:

In the corrected article, a new Acknowledgments section appeared as follows:

This paper was supported by Wonkwang University in 2022.

The correction will appear in the online version of the paper on the JMIR Publications website on November 1, 2023, together with the publication of this correction notice. Because this was made after submission to PubMed, PubMed Central, and other full-text repositories, the corrected article has also been resubmitted to those repositories.

